# Peer reviewers – The gate keepers of science

**DOI:** 10.4103/0970-1591.57897

**Published:** 2009

**Authors:** Nitin S. Kekre

**Affiliations:** Department of Urology, Christian Medical College, Vellore - 632 004, Tamilnadu, India. E-mail: editor@indianjurol.com

The Indian Journal of Urology under the aegis of Urological Society of India conducted the first Peer Review Workshop on October 23-24, 2009. A total of 22 members participated in this exercise. The objective of this exercise was to train the members to critically evaluate manuscripts. The enthusiasm of the participants and the faculty was very satisfying. The Indian Journal of Urology has been following the practice of peer review for the last couple of years. One may sometimes question the wisdom of the peer reviewer as many times their views do not agree with the authors. The process of peer review is not without fallacies and has not been scientifically proven. Many a times the classic papers or scientific content has not been accepted by the peers of their times but have stood the test of time. Still peer review of scientific manuscripts is a corner stone of modern science and medicine. Scientific journals rely on the objective review by the knowledgeable researchers to ensure the quality and standard of papers which they publish. Authors too learn to write better when they go through the advice or criticism of reviewers. Most of the well known high standard journals like Nature, Lancet, and BMJ rely on the process of peer review. The objective of the whole exercise to get impartial judgment, prevent duplication or malpractices in the field of publication.

So naturally, the peer reviewers play a very important part in scientific publications. They are like silent backstage workers who receive no credit for their hard work, as most journals do not offer any financial incentive for peer reviewer. A few journals, including as IJU acknowledge all the reviewers by printing their names in the final issue of each year. No wonder they are called the Gate Keepers of Science.

There is no formal teaching to peer review. Most of us learn by trial and error. And this becomes a significant handicap for most of us whenever we are asked to review a manuscript. Some departments may have a mentor who may guide the junior faculty to learn the basics of peer review. In this workshop we attempted to provide a basic understanding about the process of journal publication and peer review. The participants were introduced to the important topics by didactic lectures followed by hands on manuscript review and they were encouraged to write a critical review.

The most important requirement to become a good reviewer is to be knowledgeable on his subject; he should be willing to spend time, be honest and independent. He should keep up his commitment to the time as prescribed by the editor. He should never forget that he is the central and most important component in the process of publication and has tremendous obligation both to authors and editors.

I sincerely hope that continuing the training process we will be able to generate a local resource both in medical writing and in peer review, which would help raise the standard of the journal.

I am thankful to the Urological Society of India for supporting the editorial team in this endeavor.

This issue has number of impressive articles on renal cell carcinoma and full symposium edited by Dr. Mahesh Gaitonde. He is the Clinical Director at University of Cincinnati College of Medicine, Cincinnati, Ohio, USA.

Nephron sparing surgery is rapidly becoming the standard of care for incidentally detected SOL. But it is important to pause and think that despite the huge number of partial nephrectomies carried out in the last decade, the mortality from RCC hasn't declined in the same proportion. It highlights that significant number of patients undergo partial nephrectomies without apparent benefit. Approximately 15-20% of these lesions turn out to be benign. Hence there is an urgent need for basic science and research to be able to distinguish between the good, the bad and the ugly. I am thankful to Dr. Gaitonde and his co-authors for this indepth symposium on renal cell carcinoma.

As 2009 draws to a close, IJU has achieved a mile stone of being available in PubMed. I take this opportunity to thank my co-editors for their hard work and the entire team of Medknow Publications for their support.

I wish you all a very happy and Prosperous 2010,

See you all at Agra.

With best wishes.

